# RNA-seq Brings New Insights to the Intra-Macrophage Transcriptome of *Salmonella* Typhimurium

**DOI:** 10.1371/journal.ppat.1005262

**Published:** 2015-11-12

**Authors:** Shabarinath Srikumar, Carsten Kröger, Magali Hébrard, Aoife Colgan, Siân V. Owen, Sathesh K. Sivasankaran, Andrew D. S. Cameron, Karsten Hokamp, Jay C. D. Hinton

**Affiliations:** 1 Department of Microbiology, School of Genetics and Microbiology, Moyne Institute of Preventive Medicine, Trinity College, Dublin, Ireland; 2 Institute of Integrative Biology, University of Liverpool, Liverpool, United Kingdom; 3 Department of Biology, University of Regina, Regina, Saskatchewan, Canada; 4 Department of Genetics, School of Genetics and Microbiology, Smurfit Institute of Genetics, Trinity College, Dublin, Ireland; Univeristy of Washington School of Medicine, UNITED STATES

## Abstract

*Salmonella enterica* serovar Typhimurium is arguably the world’s best-understood bacterial pathogen. However, crucial details about the genetic programs used by the bacterium to survive and replicate in macrophages have remained obscure because of the challenge of studying gene expression of intracellular pathogens during infection. Here, we report the use of deep sequencing (RNA-seq) to reveal the transcriptional architecture and gene activity of *Salmonella* during infection of murine macrophages, providing new insights into the strategies used by the pathogen to survive in a bactericidal immune cell. We characterized 3583 transcriptional start sites that are active within macrophages, and highlight 11 of these as candidates for the delivery of heterologous antigens from *Salmonella* vaccine strains. A majority (88%) of the 280 *S*. Typhimurium sRNAs were expressed inside macrophages, and SPI13 and SPI2 were the most highly expressed pathogenicity islands. We identified 31 *S*. Typhimurium genes that were strongly up-regulated inside macrophages but expressed at very low levels during *in vitro* growth. The SalComMac online resource allows the visualisation of every transcript expressed during bacterial replication within mammalian cells. This primary transcriptome of intra-macrophage *S*.-Typhimurium describes the transcriptional start sites and the transcripts responsible for virulence traits, and catalogues the sRNAs that may play a role in the regulation of gene expression during infection.

## Author Summary

The burden of Salmonellosis remains unacceptably high throughout the world and control measures have had limited success. Because *Salmonella* bacteria can be transmitted from the wider environment to animals and humans, the bacteria encounter diverse environments that include food, water, plant surfaces and the extracellular and intracellular phases of infection of eukaryotic hosts. An intricate transcriptional network has evolved to respond to a variety of environmental signals and control the “right time/ right place” expression of virulence genes. To understand how transcription is rewired during intracellular infection, we determined the primary transcriptome of *Salmonella enterica* serovar Typhimurium within murine macrophages. We report the coding genes, sRNAs and transcriptional start sites that are expressed within macrophages at 8 hours after infection, and use these to infer gene function. We identified gene promoters that are specifically expressed within macrophages and could drive the intracellular delivery of antigens by *S*. Typhimurium vaccine strains. These data contribute to our understanding of the mechanisms used by *Salmonella* to regulate virulence gene expression whilst replicating inside mammalian cells.

## Introduction

*Salmonella enterica (S*. *enterica)* is a food and water-borne pathogen responsible for widespread disease in humans and other animals. The serovars responsible for typhoid fever kill more than 250,000 people per year, while an estimated 94 million cases of *Salmonella*-mediated gastroenteritis cause 155,000 deaths each year [1,2]. Recently, it has been discovered that non-typhoidal serovars are causing an epidemic of invasive disease that is killing 680,000 people each year [3].

Decades of intense research have revealed intricate details of *Salmonella* pathogenicity [4]. *S*. *enterica* initiates infection in the small intestine by penetrating the mucus layer that protects the gut epithelium. During the infection process, *S*. *enterica* endures a series of hostile environments within the host, including the acidity of the stomach, antimicrobial peptides and bile in the intestine, and the toxicity of intracellular vacuoles [5]. These challenges are met by physiological and metabolic adaptations that allow the bacterium to resist the innate host defences. *Salmonella* pathogenicity island (SPI) 1 and SPI4-encoded proteins, and other virulence determinants, mediate the entry into epithelial cells [4,6]. The bacteria subsequently exit from epithelial cells and are taken up by the phagocytic cells of the innate immune system such as macrophages [7,8].

*S*. *enterica* responds to the phagosomal environment within macrophages by secreting effector proteins that generate a specialized intracellular compartment, the *Salmonella*-containing-vacuole (SCV). The SCV allows *S*. *enterica* to evade macrophage killing, and infected macrophages become a vehicle for systemic bacterial spread [9,10]. Physiological, metabolic and effector protein-mediated adaptation strategies allow the bacteria to replicate within the SCV, and to form persister cells [10,11]; many of these adaptive processes are regulated at the transcriptional level [12].

Bacterial gene regulation is mediated by a combination of transcription factors, nucleoid-associated proteins and regulatory small non-coding RNAs (sRNAs). Following the publication of the first *S*. *enterica* genome, microarray-based transcriptomic approaches were used to define regulons and stimulons of the model pathogen *S*. *enterica* serovar Typhimurium (*S*. Typhimurium) [13]. Because the microarray-derived data only provided a limited view of *Salmonella* gene expression inside macrophages [14–16], an RNA-seq-based approach was required to gain the information for understanding mechanisms of gene regulation. RNA-seq analysis generates high-resolution transcriptomic data and accurate information on gene expression levels, and provides extensive information concerning the location of Transcriptional Start Sites (TSS), the 5′ and 3′ un-translated regions of genes, antisense transcription, and sRNAs. We recently used this approach to reveal the complete transcriptional network of *S*. Typhimurium during growth in 22 laboratory conditions [17].

Here, we present the primary transcriptome of intra-macrophage *S*. Typhimurium strain 4/74. All intra-macrophage gene expression and transcriptional organisation data are presented in our online resource, SalComMac [http://tinyurl.com/SalComMac].

## Results and Discussion

### The primary transcriptome of intra-macrophage *S*. *Typhimurium*

**Fig 1:**
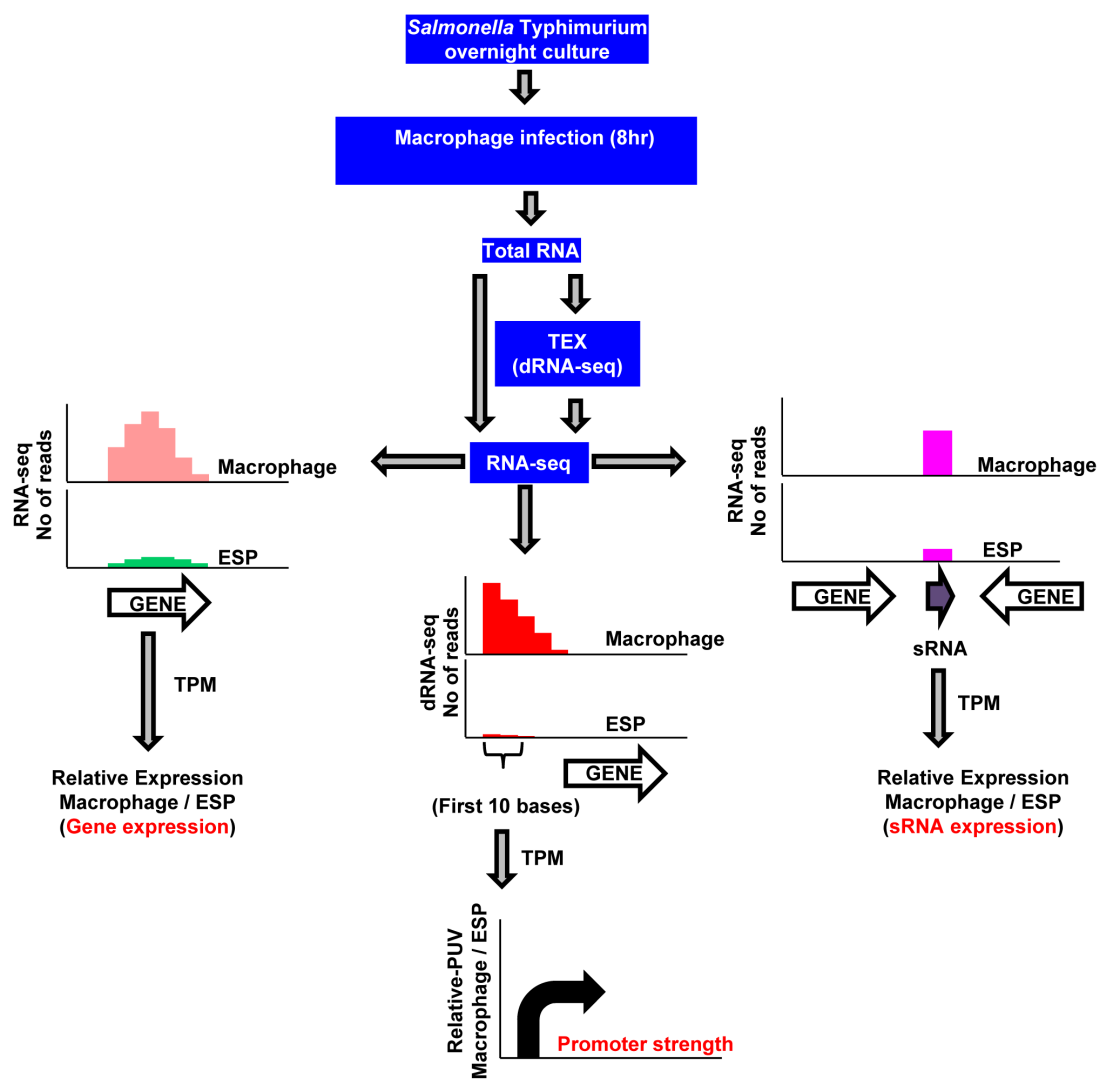
RNA-seq–based strategy to identify promoters, transcribed regions, and small RNAs of *S*. Typhimurium active during macrophage infection. *S*. Typhimurium strain 4/74 was grown within macrophages for 8 hours using the gentamicin protection assay, and bacterial RNA was isolated using TRIzol (Materials and Methods). The cDNA generated from total RNA was sequenced either directly for gene/sRNA expression analysis (RNA-seq) or after enrichment of primary transcripts (dRNA-seq), and compared with data from 4/74 grown to ESP [17]. The graphs show representations of sequence reads mapped uniquely against the 4/74 genome in different conditions. Transcript per Million (TPM) analysis was used to calculate gene expression values from the number of sequence reads mapped against the 4/74 genome. The promoter usage value (PUV) indicates the TPM value of the first 10 nucleotides from the transcription start sites (TSS) in the direction of transcription, and represents promoter strength. Each curved arrow indicates location of TSS upstream of the respective gene; the width and height of each curved arrow is proportional to TSS expression, based on relative PUV, macrophage versus ESP.

The intra-macrophage transcriptome of *S*. *enterica* was determined with *S*. Typhimurium strain 4/74 (Dataset 1 in S1 Table) within cultured murine RAW 264.7 macrophage-like cells that do not express the Nramp1 (Slc11a1) host resistance cation-efflux pump [18]. Because earlier transcriptomic analyses showed that more than 90% of *S*. Typhimurium genes were expressed at similar levels during early, middle and late stages of macrophage infection [14], we focused on a single time point. We used eight hours post-infection to coincide with the nitrosative burst in *Salmonella*-infected murine macrophages [14]. Total bacterial RNA was isolated and analysed by RNA-seq [17] (Materials and Methods) (Fig 1). Overall, 136 million sequence reads were generated from seven cDNA libraries. These represent two biological replicates of intra-macrophage *Salmonella* RNA-seq, two biological replicates of intra-macrophage *Salmonella* differential RNA-seq (dRNA-seq) and RNA-seq of the Δ*ssrA* mutant and two biological replicates of wild-type 4/74 grown under *in vitro* SPI2-inducing conditions. Between 5 and 10 million uniquely-mapped reads were obtained from each library (Dataset 2 in S1 Table), providing sufficient coverage for robust transcriptomic analysis [19]. Gene expression values were calculated by the Transcripts Per Million (TPM) approach [20]. A threshold TPM value of 10 was used as a cut-off to define gene expression (Materials and Methods) [17]. The intra-macrophage transcriptome was compared to our published RNA-seq-based transcriptome for early stationary phase (ESP), an infection-relevant *in vitro* growth condition that is associated with high expression of *S*. Typhimurium SPI1 genes [17,21].

**Fig 2:**
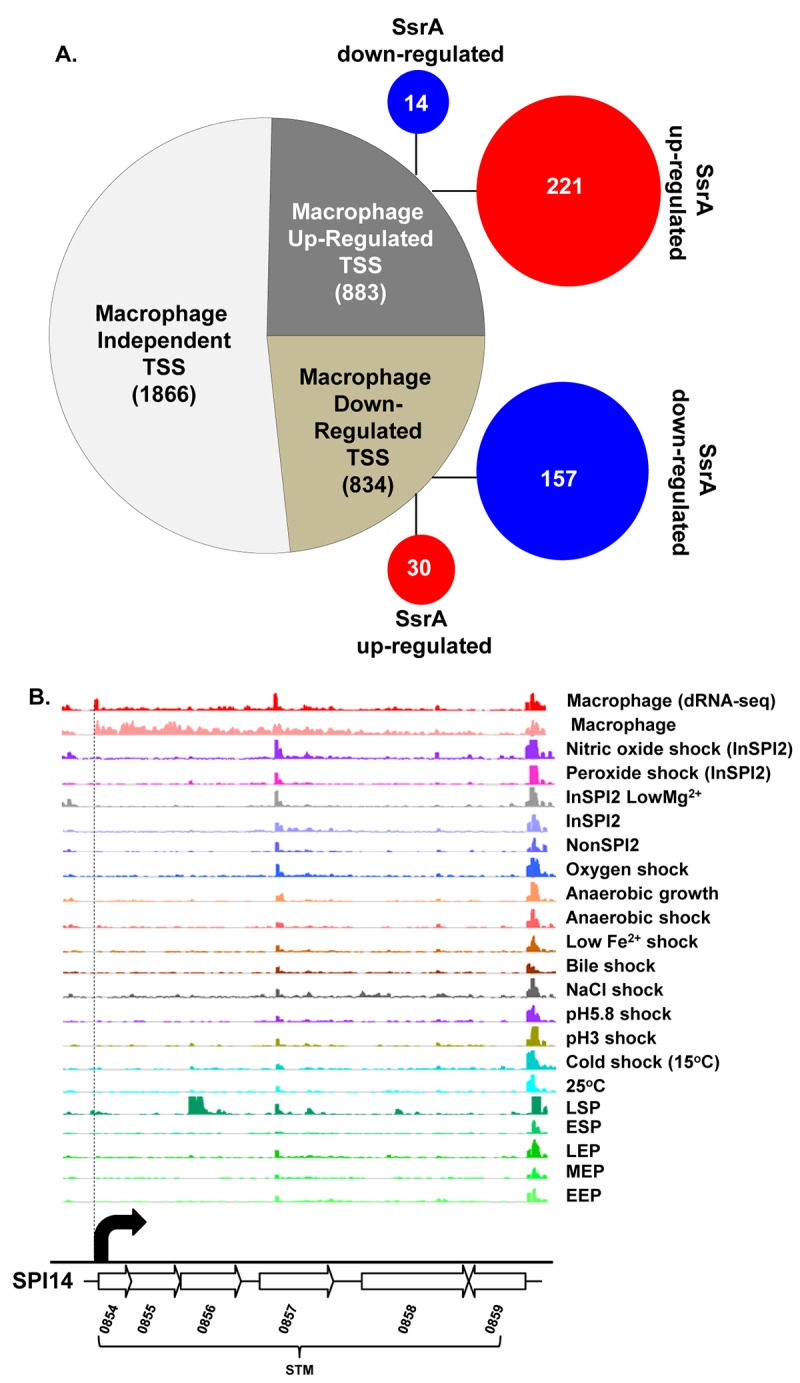
The primary transcriptome of intra-macrophage *Salmonella*. (A) Classification of 3583 *Salmonella* TSS during intra-macrophage proliferation (Dataset 3 in S1 Table). The TSS were categorized based on their relative-PUV, macrophage versus ESP (Materials and Methods). The red and blue circles represent the TSS that are up/down-regulated in the *∆ssrA* versus InSPI2 experiment, respectively (B) The STM0854 TSS (indicated by the dotted vertical line) is a representative of a TSS highly up-regulated in macrophages. Each horizontal arrow represents the gene in scale with the whole island. Each coloured track above the island represents RNA-seq/dRNA-seq reads mapped against the genome in the corresponding conditions, visualized in the IGB browser. Each curved arrow indicates the location of a TSS; the width and height of each curved arrow is proportional to the TSS expression, based on relative PUV, macrophage versus ESP (Dataset 3 in S1 Table) (Materials and Methods).

The precise nucleotide position of individual TSS was identified on a genome-wide scale by dRNA-seq [22]. In total, 3583 TSS were expressed by *S*. Typhimurium during infection of macrophages (Dataset 3 in S1 Table). This included 3538 TSS expressed in the ESP condition [17] and 45 TSS which were newly identified in this study. To assign a relative strength to each TSS we determined the expression levels of the first 10 bases of each transcript, designated the promoter usage value (PUV) [17,23]. Because >99% of *S*. Typhimurium protein coding genes have a 5’ untranslated region (UTR) and 15% of protein-coding genes possess multiple TSS, the PUV allows promoter strength to be quantified independently of gene expression [17]. We used the relative PUV to compare the expression of *S*. Typhimurium TSS between the intra-macrophage and the ESP *in vitro* condition, and categorised the TSS as either ‘Macrophage up-regulated’, ‘Macrophage down-regulated’ or ‘Macrophage-independent’ (Materials and Methods). Of the 3583 TSS expressed in macrophages, 883 were macrophage up-regulated and 834 were macrophage down-regulated, compared with ESP (Fig 2A; Dataset 3 in S1 Table). The TSS of the *lgl-ripABC* (STM3117-3120) SPI13 operon [24,25] was the most highly up-regulated, with a relative PUV of >500 -fold. Other highly up-regulated TSS controlled the expression of genes such as *trpE* and *sseJ*. We found that 72% of the promoters reported to be highly expressed in the murine spleen [26] were up-regulated in RAW macrophages.

Forty five new TSS were identified in this study, including the TSS of STM0854 that controls intra-macrophage expression of the major polycistronic transcript of SPI14 (Fig 2B). Other novel TSS controlled the expression of genes involved in several core cellular processes including *bglA*, *entB*, *fliN and nrdE*, and a TSS that initiated a transcript antisense to the *stfD* coding gene.

Of the 834 macrophage down-regulated TSS, the biggest reduction in promoter expression between macrophages and the ESP condition was more than 200-fold and associated with SPI1 genes and the flagellin-encoding *fliC* gene (Dataset 3 in S1 Table).

### The transcriptional organisation of SPI2 during infection of macrophages

**Fig 3:**
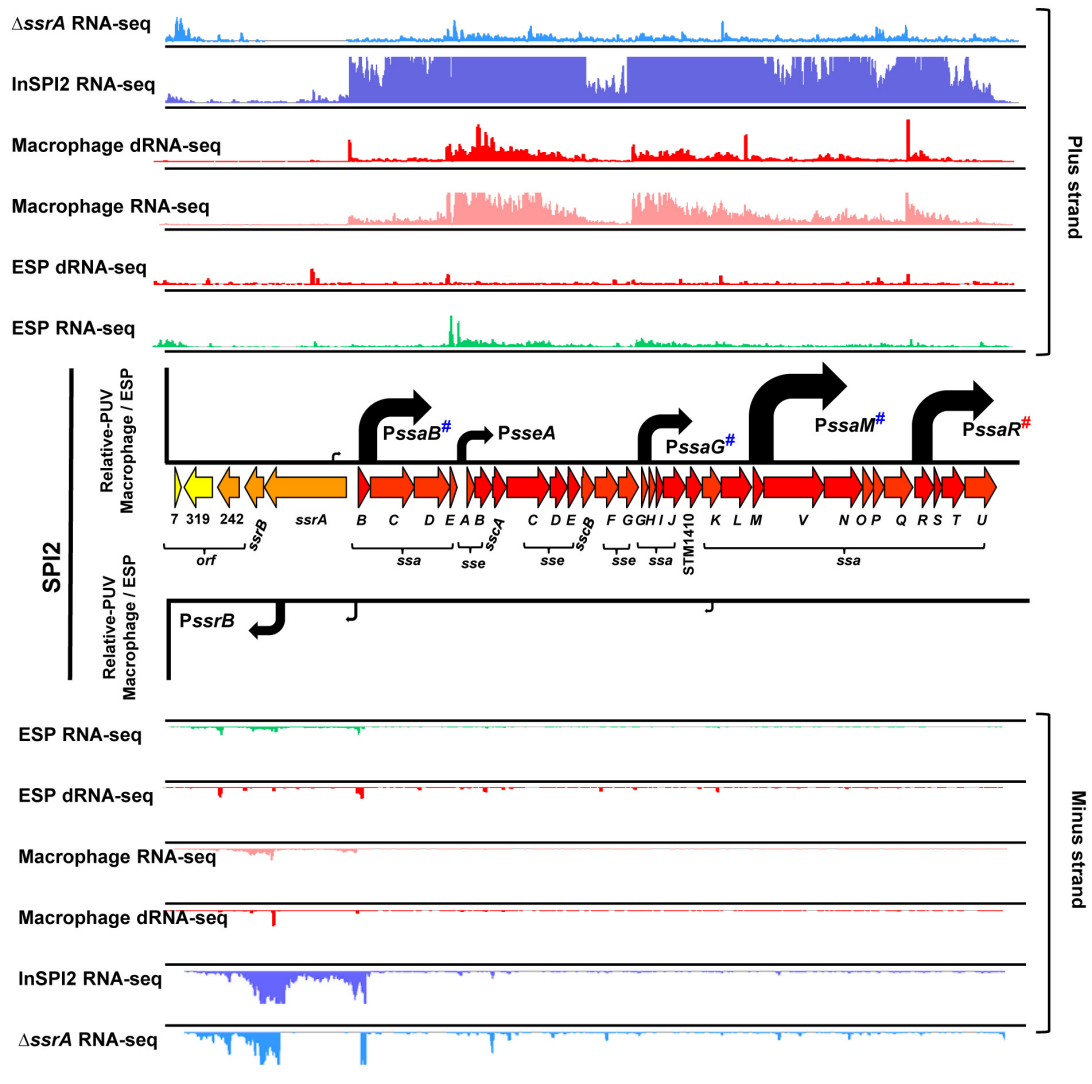
The transcriptional organization of SPI2 in intra-macrophage *Salmonella*. Horizontal arrows represent individual SPI2 genes in scale with the whole Island. The *ssrA*-*B* gene products regulate SPI2 expression, the *sseA*-*E* genes encode effector proteins, the *sscA*-*B* genes encode chaperone proteins and the *ssaB*-*E* and *ssaG*-*U* loci encode components of the T3SS apparatus. The colour of each gene represents its relative expression, macrophage versus ESP (Dataset 4 in S1 Table) based on the colour scale given in Fig 4. Each track above and below the island shows the mapping of the RNA-seq or dRNA-seq reads against the plus or minus strand of the 4/74 chromosome visualized in IGB. Each curved arrow indicates the location of a TSS upstream of the respective gene; the width and height of each curved arrow is proportional to the TSS expression, based on relative PUV, macrophage versus ESP (Dataset 3 in S1 Table) (Materials and Methods). The red hash on P*ssaR* indicates that the TSS was reported previously [17], and confirmed in this study by 5’RACE (S1 Fig). The blue hashes indicate that the location of promoters P*ssaB*, P*sseA*, P*ssaG*, P*ssaM* and P*ssrB* that have been confirmed independently by 5’RACE [107] (S1 Fig).

*Salmonella* pathogenicity island 2 (SPI2) is required for *Salmonella* replication within eukaryotic cells and for systemic infection of mammalian hosts. SPI2 encodes the type III secretion system (T3SS) that delivers many effector proteins responsible for the function of the SCV within macrophages [4,27]. The transcriptional organization of SPI2 is shown in Fig 3. We recently used dRNA-seq to discover a TSS upstream of *ssaR* [17], which we now confirm by 5’ RACE (S1 Fig). SPI2 is therefore transcribed as six operons inside macrophages (Fig 3).

All SPI2 genes were up-regulated within macrophages, reflecting the phosphate/magnesium starvation and the acidity of the SCV [28,29]. The RNA-seq data were used to calculate promoter usage values for the different SPI2 promoters, identifying P*ssaM* as the most up-regulated SPI2 promoter, followed by P*ssaR* and P*ssaB* (Dataset 3 in S1 Table). We note that each of the six SPI2 promoters was also transcribed in the “InSPI2” growth condition, confirming that expression of all SPI2 operons occurs *in vitro* when stimulated by growth in an acidic low-phosphate environment [30].

The SPI2 island and genes that encode SPI2-translocated effectors are activated by the SsrAB two component system [31]. The SsrA sensor kinase phosphorylates the SsrB response regulator to activate gene expression [32–34]. To investigate the role of SsrA in the regulation of macrophage up-regulated TSS, we used RNA-seq to analyse the transcriptome of a Δ*ssrA* mutant and wild-type 4/74 grown in InSPI2 medium. Of the 883 macrophage up-regulated TSS, 221 showed reduced (>2-fold) expression in the absence of SsrA and we infer that these are SsrA-activated (Fig 2A; Dataset 3 in S1 Table). All the genes that encode SPI2-translocated effector proteins were controlled by SsrA-activated promoters. There are 662 macrophage up-regulated TSS that appear to have SsrA-independent regulatory mechanisms, and these merit further study.

### Intra-macrophage expression of *S*. Typhimurium pathogenicity islands and effector-coding genes

**Fig 4:**
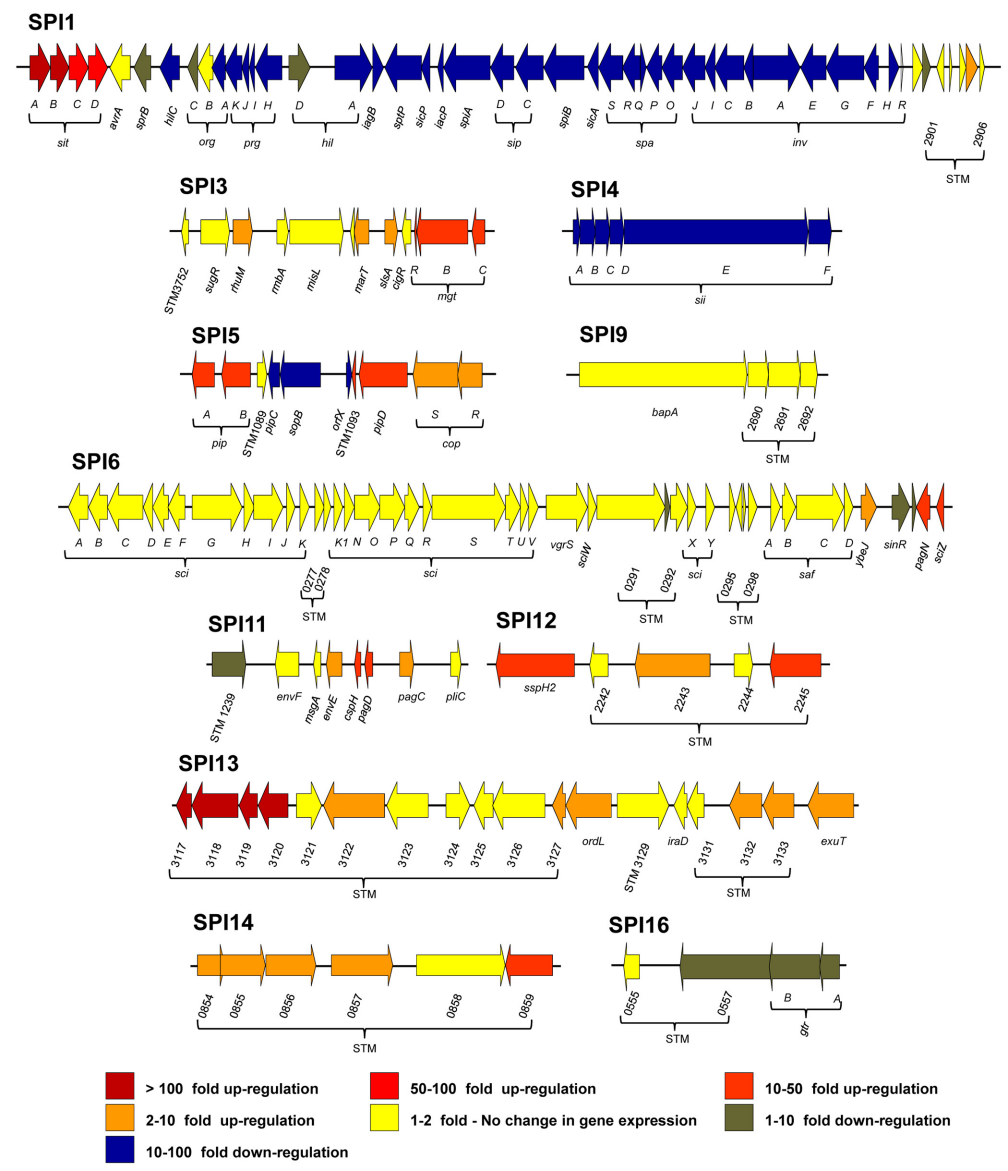
The relative intra-macrophage expression of the different pathogenicity islands of *S*. Typhimurium. Each horizontal arrow represents individual genes to scale within each SPI Island; the different islands are not scaled against each other. The colour of each arrow represents relative gene expression, macrophage versus ESP (Datasets 4 and 5 in S1 Table) based on the colour scale at the bottom of the figure. SPI2 expression is shown in Fig 3.

*S*. Typhimurium carries 12 pathogenicity islands on the chromosome of strain 4/74 [17,35,36]. Expression profiles of *S*. Typhimurium pathogenicity islands (Fig 4; Datasets 4 and 5 in S1 Table) reveal that SPI2 and SPI13 were the most highly up-regulated during infection of macrophages, by an average of 44 and 82-fold, respectively (Dataset 5 in S1 Table). The SPI3, SPI5, SPI11, SPI12 and SPI14 islands showed moderate intra-macrophage up-regulation. SPI6 and SPI9 show macrophage-independent expression, and both SPI1 and SPI4 were significantly down-regulated inside macrophages.

**Fig 5:**
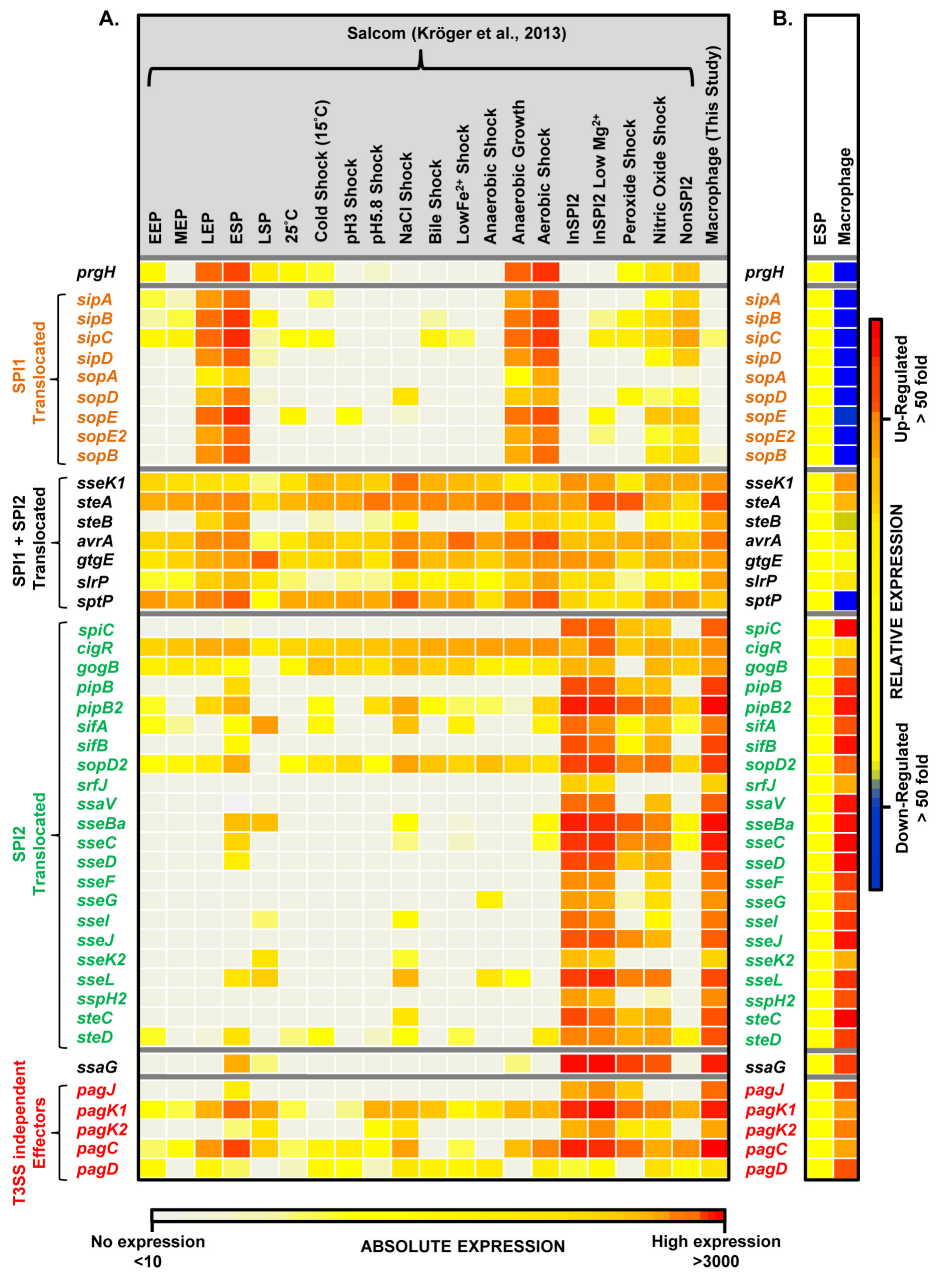
The intra-macrophage expression of *Salmonella* effector genes. (A) A comparison of absolute expression levels of different *S*. Typhimurium effector genes within macrophages (Dataset 4 in S1 Table) and in 20 *in vitro* infection-related conditions [17]. The heatmap colours represent the absolute expression levels (log_10_ TPM values) based on the colour bar, ranging from TPM values of 49 to 706. (B) The relative expression levels (macrophage versus ESP) of each gene are defined in the colour bar to the right. The genes *prgH* and *ssaG* are included to show the SPI1-like and SPI2-like patterns of expression, respectively. The T3SS-independent genes were described by Kidwai et al. (2013) [108].

Effector proteins of *S*. Typhimurium are secreted via the SPI1 T3SS, the SPI2 T3SS or through both translocation systems. We reported that the genes encoding SPI1-translocated effectors showed a SPI1-like expression pattern, and genes encoding SPI2-translocated effectors showed a SPI2-like expression pattern [17]. Our data show that the genes encoding all SPI2-translocated effectors were highly macrophage up-regulated (Dataset 4 in S1 Table) (up to 70-fold), and the genes that encode the 7 effectors that are secreted by both the SPI1 and SPI2 T3SS were all expressed inside macrophages; the TPM values range from 50 to 230 (Fig 5). In contrast, genes encoding the 9 SPI1-translocated effectors were all macrophage down-regulated, by up to 160-fold, and were not significantly expressed within macrophages. Clearly, the actual intra-macrophage expression level of genes that encode candidate effector proteins has biological relevance.

The transcriptomic data identified two specific SPI13 and SPI14-encoded operons that were highly up-regulated in macrophages (Datasets 4 and 5 in S1 Table) but were not significantly expressed in 20 *in vitro* conditions [17]. First, the SPI13-associated *lgl*-*ripABC* (STM3117-STM3120) operon was >250 fold up-regulated within macrophage. The *lgl*-*ripABC* operon is required for *Salmonella* infection [37,38], encoding enzymes that catabolise itaconate, an anti-microbial metabolite that is synthesised by infected macrophages [25,39]. Second, the SPI14-located STM0854-0857 operon is also required for *Salmonella* virulence [38], showed moderate (3 to 20-fold) intra-macrophage up-regulation, and was not expressed in *in vitro* growth conditions [17]. The TSS of the STM0854 and STM0859 transcripts were only expressed in macrophages, and not in any *in vitro* conditions. Taken together, these data suggest that the STM0854-0857 and *lgl*-*ripABC* operons respond to an intra-cellular signal that remains to be identified in macrophages. For *ripABC*, this signal may be itaconate [25].

For SPI3, the PhoP-activated *mgtCBR* operon [40] was up-regulated >15 fold within macrophages, while other SPI3 genes (*slsA*, *marT* and *rhuM*) were moderately up-regulated. The role of *mgtCBR* in virulence involves the long leader of the *mgtC* transcript that encodes MgtP. The *mgtC* leader is responsive to ATP levels [41] and inhibits F1Fo ATP synthase to maintain ATP homeostasis in the acidic intra-macrophage environment [42].

SPI5 encodes effectors translocated by both SPI1 and SPI2 T3SS [43,44]. The *sopB* gene encodes a SPI1-translocated effector and is macrophage down-regulated by 50-fold. In contrast, the gene encoding the SPI2 effector *pipB* is up-regulated. PipB localizes to the SCV membrane and brings about the formation of tubular extensions, the *Salmonella* induced filaments (SIFs) [45,46].

The SPI6-encoded Type 6 secretion system [47], is important for the colonization and systemic infections of chickens and mice [48,49]. None of the SPI6 genes were expressed in macrophages or in various *in vitro* conditions [17]. This is consistent with the reported repression of SPI6 genes by H-NS [50].

During infection of the gastrointestinal tract, the SPI1-encoded T3SS of *S*. Typhimurium is responsible for inflammatory diarrhoea and the invasion of non-phagocytic epithelial cells [51–53]. Thirty-three SPI1 genes were down-regulated within macrophages (Dataset 4 in S1 Table), and were highly expressed at ESP, confirming earlier reports [17,54]. HilA, the transcriptional activator of SPI1, is controlled by the co-ordinated action of HilC/HilD/RtsA, and consequently up-regulates the SPI1 island & SPI1-translocated genes [55–57]. The transcription of *hilA* is regulated by HilD, an important activator that controls cross-talk between SPI1 and SPI2 expression [55,58]. The *hilA*, *hilC*, *hilD* and *rtsA* regulatory genes are down-regulated more than 100-fold within macrophages, consistent with the down-regulation of the SPI1 island.

The *siiABCDEF* operon of SPI4 encodes a Type 1 secretion system, and was down-regulated within macrophages. SiiE is a non-fimbrial adhesin responsible for the adhesion of *Salmonella* to epithelial cells and is expressed during the extra-cellular phase of infection [59,60]. Cross talk between SPI1 and 4 can promote tight binding of the bacterium to the epithelial membrane, and facilitate efficient SPI1 translocation [61].

### Relating intra-macrophage gene expression to gene function

Intracellular expression of individual bacterial genes or entire regulons can be used to investigate the microenvironment inside the host cell vacuole [62]. Direct comparison between this RNA-seq-based dataset (Dataset 4 in S1 Table) and previous microarray-based transcriptomic results confirm and extend key findings from Eriksson *et al*. (2003) and Hautefort *et al*. (2008) [14,15]. The datasets all show that the most highly macrophage up-regulated *Salmonella* gene is *asr* (STM1485), required for the intra-cellular replication of *Salmonella* [63]. The 890-fold up-regulation of *asr* reflects the acidic conditions within the SCV [64] (Dataset 4 in S1 Table).

**Fig 6:**
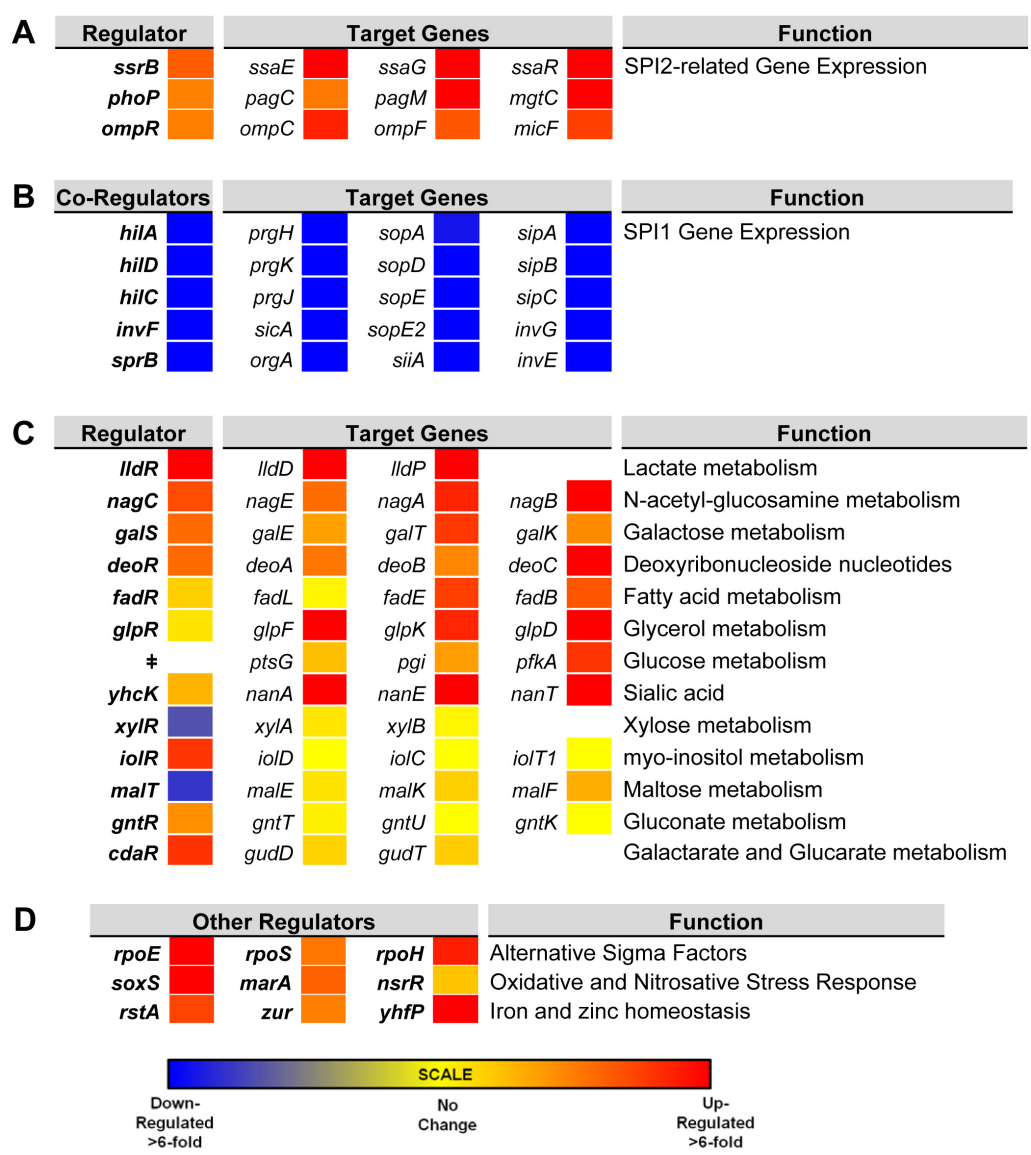
Relative intra-macrophage expression of *Salmonella* transcription factors and selected target genes. The expression of individual genes is shown as fold change, intra-macrophage versus ESP. Transcription factors are shown in bold. Target genes controlled by individual transcription factors are shown in the same row (A and C). Expression of transcription factors that regulate SPI2-related genes (A). The regulation of SPI1 genes is controlled by a hierarchy, and the transcription factors are depicted as co-regulators, with their combined target genes (B). Relative expression of 13 metabolic systems (C). Relative expression of up-regulated alternative sigma factors and transcription factors that control oxidative stress, and iron and zinc homeostasis (D). ^ǂ^No dedicated transcription factor for glucose metabolism was assigned.

To investigate the gene expression network of intra-macrophage *Salmonella*, we focused on 157 transcriptional regulators (Dataset 6 in S1 Table). The levels of 34 transcription factors were >3-fold macrophage up-regulated, and 7 transcription factors were >3-fold macrophage down-regulated. To determine whether the differential expression of individual regulators was reflected by up- or down-regulation of the associated regulons, we compared the expression of several genes controlled by each transcription factor in ESP and macrophages. We observed that the up-regulation of SPI2 regulators *ssrB*, *ompR* and *phoP* and down-regulation of SPI1 regulators *hilD*, *hilA*, *hilC*, *invF* and *sprB* correlates with the expression of their respective regulons (Fig 6A and 6B). The macrophage up-regulation of regulons that detoxify peroxide, detoxify nitric oxide and relieve envelope stress and protein misfolding (*soxS*, *oxyR*, *marA*, *marS*, *rpoE*, *rpoH* and *nsrR* regulons and genes *hmpA*, *msrA*, *ycfR*, *sbp*, *sodC*, *katG*), reflects the bacterial response to the oxidative and nitrosative bursts that occurred during the infection process.

Bacterial genes were assigned to functional groups to investigate the metabolic resources of macrophages. The most up-regulated functional categories of *S*. Typhimurium genes within macrophages are involved in carbohydrate and amino acid metabolism (S2 Fig). The “nutritional immunity” hypothesis posits that the innate immune response of the host reduces the availability of important nutrients for intracellular bacteria [65], which may explain why *S*. Typhimurium has evolved the ability to utilise a diverse range of host nutrients, including some sugars and amino acids that accumulate in murine macrophages during intracellular infection [66]. It is known that the major carbon sources utilised by *S*. Typhimurium in macrophages of the mouse spleen are deoxyribonucleotides, fatty acids, glucose, gluconate, glycerol, lactate and N-acetyl-glucosamine [67]. In our study, we observe the concerted up-regulation of multiple metabolic regulons in RAW macrophages that are consistent with the simultaneous degradation of deoxyribonucleotides, fatty acids, galactose, glucose, gluconate, glycerol, lactate, N-acetyl-glucosamine and sialic acid, while regulons controlling gluconate, maltose, *myo*-inositol and xylose metabolism showed significant macrophage down-regulation (Fig 6). Our current understanding of the intracellular metabolism of *Salmonella* in cultured macrophages coupled with the comprehensive data available for S. Typhimurium during infection of the murine spleen [66] suggest that cultured macrophages represent a good model for the study of the intracellular metabolism of *Salmonella*.

Mammalian macrophages reduce intracellular levels of metals such as iron as part of their strategy to limit bacterial replication [68], and *S*. Typhimurium responds by switching on the expression of metal-uptake systems. These include the intra-macrophage up-regulation of the *sitABCD* operon, responsible for manganese and iron transport [69] and of genes responsible for iron transport and biogenesis of iron-sulfur cluster containing proteins (*ent*, *fep*, *fhu*, *iro*, *sfb*, *sit* and *suf* genes, as well as the *yhfP* (*iscR*), and *rstA* regulons), magnesium (*mgtCBR*) transport and zinc (*zur*) uptake. We suggest that these expression patterns reflect the relatively low levels of magnesium, manganese, iron and zinc metals within the SCV [70]. Genes encoding the flagella and chemotaxis systems were significantly down-regulated in macrophages (between 50 to 100-fold), consistent with previous reports for both the Typhimurium and Typhi serovars [14,15,71] (Dataset 4 in S1 Table; S2 Fig). Specifically *flh*, *flg*, *fli*, *flj*, *mot*, *che* and *aer* genes were down-regulated. The *flhDC-*mediated regulation of flagellar transcription is complex [72], and cross-talk between SPI1 and flagellar genes was recently reported [73]. The flagellar regulator FliZ is a post-transcriptional activator of *flhDC* that positively regulates SPI1 by activating the *hilD-rtsAB* cascade [74]. In turn, RtsB represses the *flhDC* promoter [57]. These regulatory mechanisms probably account for the down-regulation of flagellar genes within macrophages, consistent with the shut-down of flagellar synthesis associated with the non-motile bacteria found in the SCV [75]. This contrasts with the reported up-regulation of SPI1 and flagella that occurs when *S*. Typhimurium encounters the cytosol of epithelial cells [75].

### Thirty one *Salmonella* genes are specifically up-regulated within macrophages

**Fig 7:**
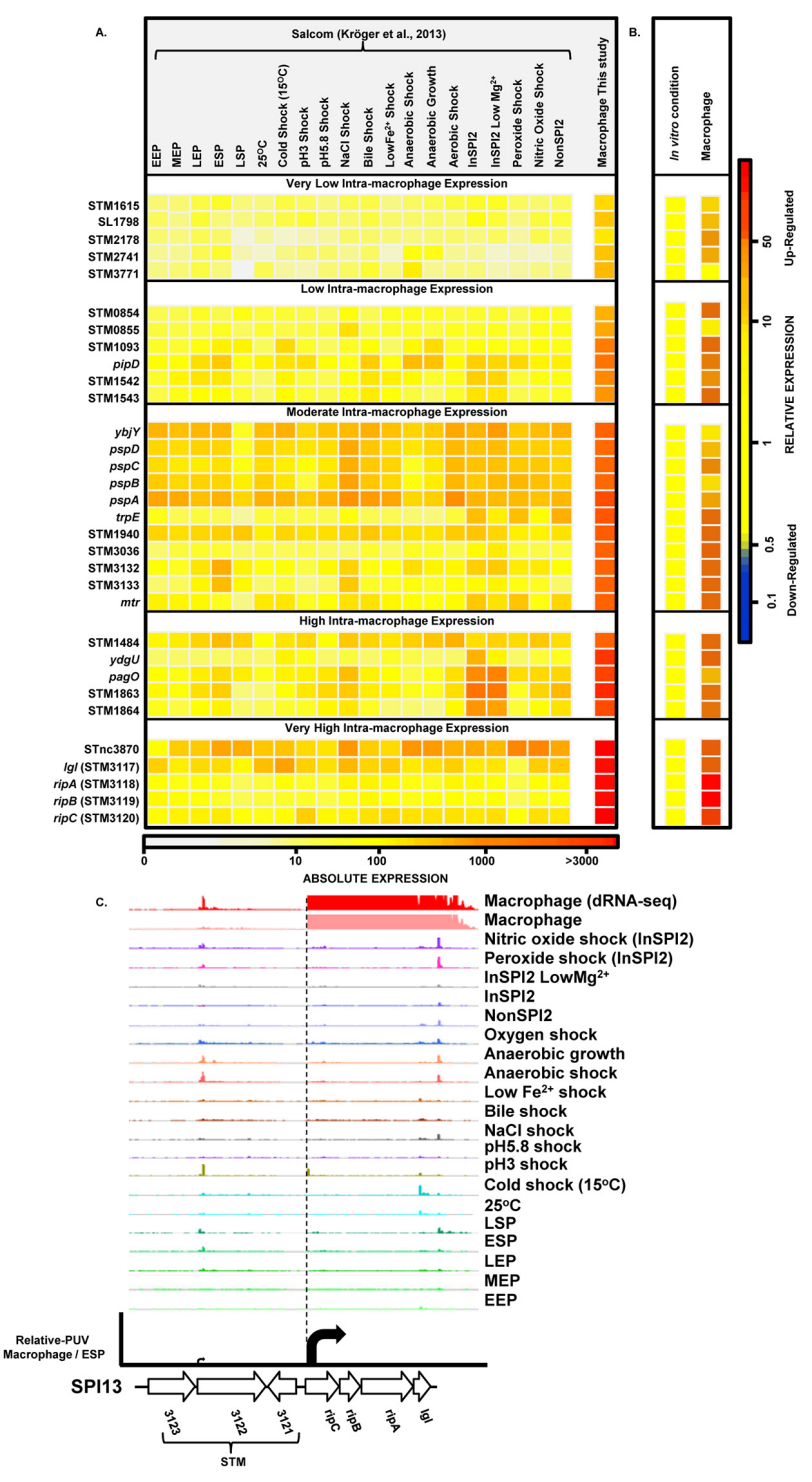
*Salmonella* genes that are specifically up-regulated inside macrophages. (A) Heatmap showing expression of 31 *S*. Typhimurium genes that are specifically up-regulated during infection of macrophages, compared to 20 *in vitro* conditions [17] (Dataset 7 in S1 Table) (Materials and Methods). The heatmap colours represent the absolute expression levels (log_10_ TPM values) based on the colour bar below. (B) The relative expression level of each gene is the fold-change of macrophage versus [expression in the *in vitro* condition where the gene in maximally expressed], based on the colour bar to the right. (C) The SPI13 operon (*lgl* [37] and *ripABC* [25], or STM3117 and STM3118-STM3120, respectively) is highly induced within macrophages.

To find genes that were up-regulated in the intra-macrophage environment but not in standard laboratory conditions, we used a comparative transcriptomic approach to identify genes that showed significantly higher expression in macrophages than in any of 20 *in vitro* conditions [17] (Materials and Methods). Our analysis identified 31 genes that were specifically up-regulated within macrophages (Dataset 7 in S1 Table; Fig 7A and 7B). These represent an interesting class of bacterial genes that are up-regulated in macrophages due to a factor encountered within macrophages and not in the *in vitro* growth conditions. The STM3117-STM3120 (*lgl-ripABC*) genes are a good example, of highly macrophage-induced genes (Fig 7C) that are involved in the detoxification of two SCV-specific metabolites, methylglyoxal and itaconate [24,25]. We propose that comparative transcriptomics will be a useful approach for identifying genes that respond to specific components of the SCV environment. The majority of the genes in Fig 7A have a STM or a *yxx* prefix and are designated as “FUN” genes, for “function unknown” [76]. Overall, 18 of the 31 genes that were specifically up-regulated within macrophages have previously been shown to be required for virulence (Dataset 7 in S1 Table). We speculate that these FUN genes respond to a specific component of the intra-vacuolar environment of the macrophage and could play important roles in the process of infection.

### *Salmonella* promoters with potential therapeutic applications

The identification of a discrete set of promoters that are up-regulated in macrophages could have therapeutic applications. Attenuated strains of *S*. Typhimurium have been used extensively as vaccines [77], and for expressing anti-cancer proteins within tumours [78]. These technologies require specific *Salmonella* gene promoters to drive the production of foreign antigens [79]. For example, the *ssaG* promoter of SPI2 has been used to express *E*. *coli* heat labile toxin in *S*. Typhimurium [80]. However, the *ssaG* promoter is active in the gut [81,82], and so may not be the ideal antigen delivery system. We sought to identify candidate promoters with the characteristics required to deliver antigens from attenuated live vaccine strains of *S*. Typhimurium during intracellular infection.

We screened our intra-macrophage promoter expression data to identify primary TSS that were highly expressed within macrophages, and driving a downstream gene that was highly macrophage up-regulated. Eleven promoters were identified as suitable for antigen delivery during infection (Dataset 8 in S1 Table), controlling the *asr*, *bioB*, *iroB*, *sseJ*, STM0854 (SPI14) and *ripC* (SPI13) genes. Of these, *sseJ* is highly expressed within mouse organs [83]. The *ripC* promoter may be ideal for antigen delivery as it is highly and specifically induced inside macrophages (Dataset 3 in S1 Table; Fig 7C). However, high-level expression of heterologous antigens does not always generate the optimal stimulation of immune responses [79], and over-expression of certain proteins could compromise bacterial fitness. For this reason, we categorized the macrophage-up-regulated genes from Fig 7A, based on their levels of intra-macrophage expression and identified the promoter of STM0854 as a promising candidate for moderate but specific induction of gene expression within macrophages (Fig 7B). The 11 promoter candidates have the potential to deliver different levels of heterologous antigens and could be used to improve *Salmonella*-based intracellular vaccine delivery systems.

### The sRNA transcriptome of intra-macrophage *Salmonella*

**Fig 8:**
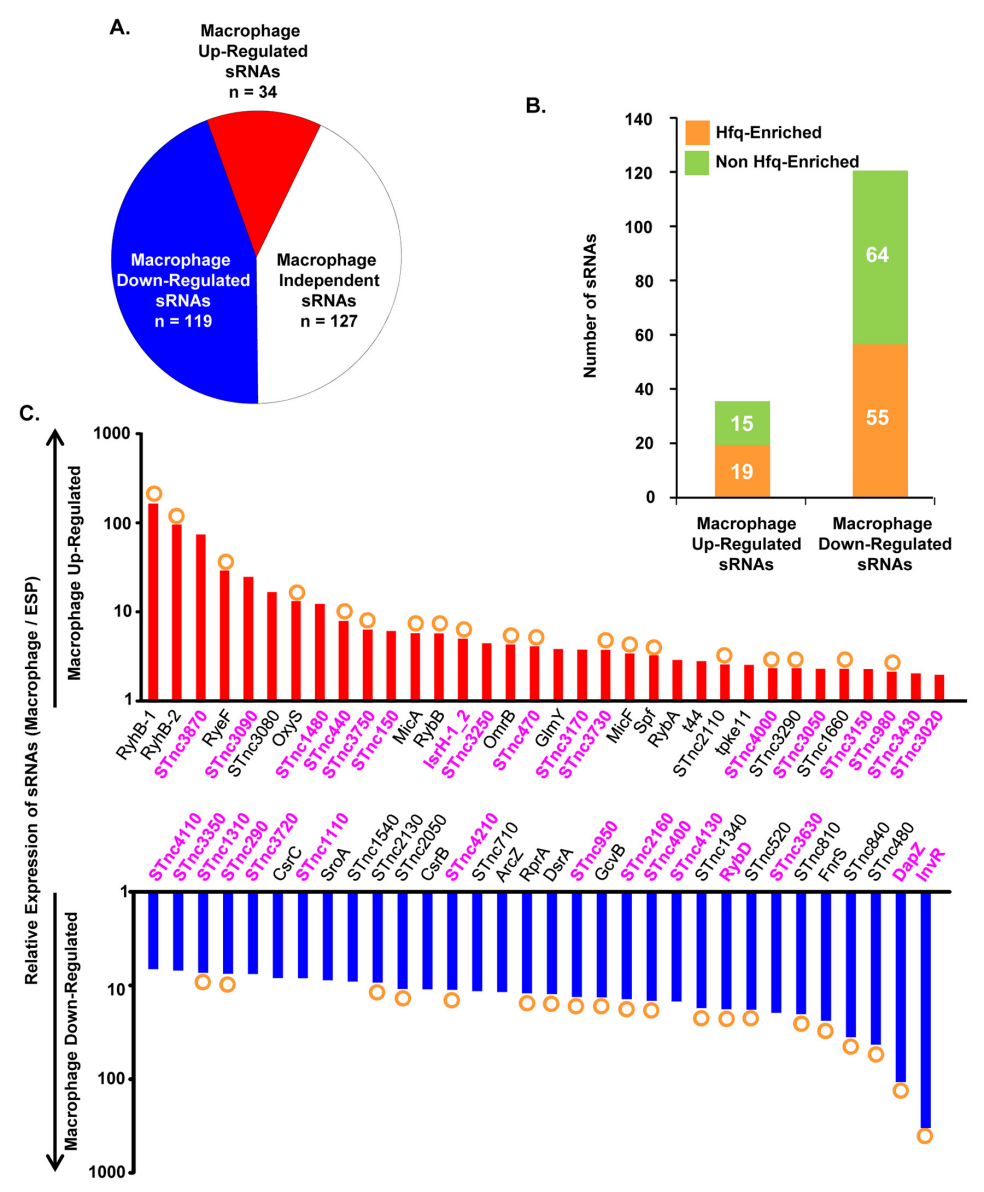
Intra-macrophage expression profile of *Salmonella* sRNAs. (A) Total number of sRNAs that are up or down-regulated in macrophages, based on the relative TPM value (>2-fold macrophage versus ESP; Dataset 9 in S1 Table). (B) Histograms representing the total number of Hfq-associated sRNAs [109] that are up or down-regulated within macrophages. (C) The relative expression of *S*. Typhimurium sRNAs in macrophages (TPM values; macrophage versus ESP). The red histograms depict the 34 macrophage up-regulated sRNAs (>2-fold). The blue histograms show sRNAs down-regulated >6-fold within macrophage (Dataset 9 in S1 Table). The orange circles indicate Hfq-bound sRNAs [17]. The names of sRNAs shown as magenta bold text are specific to the *Salmonella* genus (Dataset 10 in S1 Table).

Bacterial gene expression is controlled by transcription factors, nucleoid-associated proteins and sRNAs. Bacterial sRNAs are roughly 50–300 nucleotides in length, and play regulatory roles in key physiological activities like iron homeostasis, carbon metabolism, anaerobic adaptation, envelope stress and pathogenesis [84–88]. To date, 280 sRNAs have been identified in *S*. Typhimurium 4/74 [17], but little is known about their role in virulence [5,85]. The fact that 246 of 280 sRNAs were expressed within macrophages (TPM value >10; Dataset 9 in S1 Table) suggests that many could potentially play a regulatory role during infection. In terms of relative expression, we found that 34 sRNAs were macrophage up-regulated and 119 sRNAs were macrophage down-regulated, compared to ESP (Dataset 9 in S1 Table; Fig 8A). The Hfq chaperone protein mediates sRNA-mRNA interactions and binds to at least 115 *S*. Typhimurium sRNAs [17,89], of which 19 were up-regulated within macrophages (including RyhB-1/2, OxyS, MicF and RybB) and 56 were down-regulated (including ArcZ, DsrA and DapZ), compared to ESP (Dataset 9 in S1 Table; Fig 8B).

The expression patterns of well-characterised sRNAs provide insight into the conditions experienced by *S*. Typhimurium bacteria in the SCV. For instance, up-regulation of the RpoE-dependent sRNAs MicA and RybB inside macrophages likely reflects envelope stress of *S*. Typhimurium during intracellular proliferation [90,91]. Another sRNA that is RpoE-dependent in *E*. *coli*, MicL (RyeF) [92] is up-regulated 30-fold within macrophages, but it is not yet known whether this sRNA is controlled by RpoE in *Salmonella*. The iron-regulated homologs RyhB-1 and RyhB-2 were the most highly up-regulated sRNAs within macrophages compared to ESP (Dataset 9 in S1 Table, Fig 8C), reflecting the iron-limited intra-macrophage environment [14,17,93,94]. RyhB-1 and RyhB-2 (named RfrA and RfrB in *S*. Typhi) are also known to be important for replication of *S*. Typhi within macrophages [95]. Our data confirm that the IsrH, RyhB-1 and RyhB-2 (IsrE) sRNAs are up-regulated, as originally reported within J774 macrophages [93]. We analysed the expression of six sRNAs that were up-regulated within fibroblasts, a cell type that does not support the replication of *Salmonella* [96]. Two of these sRNAs, RyhB-1 and RyhB-2, were also up-regulated in macrophages (Dataset 9 in S1 Table). We identified several uncharacterized Hfq-associated sRNAs that were up-regulated within macrophages, including STnc440, STnc470 and STnc3750 which have an expression pattern consistent with a role in virulence. The function of these sRNAs is currently under investigation.

To determine whether macrophage-regulated sRNAs were phylogenetically conserved between fourteen serovars that represent much of the diversity of the *Salmonella* genus, we analysed 29 enterobacterial genomes (Dataset 10 in S1 Table). We found that 176 sRNAs were conserved (>90% sequence identity) within the *Salmonella* genus, but not in other members of the *Enterobacteriaceae* (<70% sequence identity), and were designated *Salmonella*-specific. About 10% (17) of the *Salmonella*-specific sRNAs were up-regulated within macrophages (including STnc440 and IsrH) while 74 were down-regulated in macrophages (including DapZ and InvR), compared to ESP (Fig 8C, Dataset 10 in S1 Table). We propose that some of these 91 macrophage-regulated sRNAs could play important roles in the regulation of gene expression during the intracellular phase of *Salmonella* infection.

### Perspective

*Salmonella* bacteria are exposed to multiple stressors within the vacuolar compartment of macrophages, including acid pH, reactive oxygen and reactive nitrogen species. Adaptation to this hostile environment has a profound impact upon the transcriptome of *S*. Typhimurium, and we have now defined the TSS and sRNAs that react to the intra-vacuolar environment during the intracellular phase of the *Salmonella* infection cycle. Our data provide an overall view of sRNA expression within macrophages, and represent a resource for the investigation of post-transcriptional regulation during the intracellular life of *Salmonella*.

This study offers new insights into the interaction of *Salmonella* with mammalian cells, and brings us a step closer to understanding the gene regulatory mechanisms that facilitate the success of this dangerous pathogen. The SalComMac online resource [http://tinyurl.com/SalComMac] is intended to simplify the comparison of the transcriptome of intra-macrophage and *in vitro* grown *S*. Typhimurium.

## Materials and Methods

### Bacterial strains, macrophage cells and growth conditions

*Salmonella enterica* subspecies *enterica* serovar Typhimurium strain 4/74 was used for all experiments; 4/74 is the prototrophic parent of strain SL1344; the two strains differ by just eight single nucleotide polymorphisms [21,35,97]. For *in vitro* RNA isolation, bacterial cells were grown overnight in 5 mL Lennox (L-) Broth (Dataset 1 in S1 Table), diluted 1:1000 into 25 mL L-broth, grown at 220 rpm and 37°C in a 250 mL flask until early stationary phase (ESP, OD_600_ 2.0) [17]. InSPI2 minimal media was used to induce expression of SPI2 *in vitro* [30]. For all intracellular studies, RAW 264.7 (ATCC) murine macrophage cells were maintained in Dulbecco’s Minimal Essential Medium (DMEM) supplemented with 5% fetal bovine serum & L-glutamine (2 mM final concentration) and MEM non-essential amino acids without antibiotics, incubated at 37°C in 5% CO_2_. All tissue culture reagents were supplied by Lonza.

### RNA isolation from intracellular *Salmonella*

Approximately 10^9^ RAW 264.7 macrophage cells were seeded in 175 cm^2^ tissue culture flasks and infected with complement-opsonized 4/74 cells at a multiplicity of infection (MOI) of 100:1 (bacteria:macrophages) [14]. Mouse serum (Charles River Laboratories) was used for opsonisation, and was stored at −80°C prior to use. After 30 minutes of infection, extracellular bacteria were killed by media containing 100 μg mL^−1^ gentamicin and incubated for a further 1h. The medium was then changed to ‘maintenance media’ containing 10 μg mL^−1^ gentamicin for the rest of the experiment. At 8 hours post infection, the infected macrophages were lysed in ice cold ‘RNA stabilisation solution’ [0.2% SDS, 19% ethanol, 1% acidic phenol in water] and incubated on ice for 30 minutes [14] to prevent RNA degradation [98,99]. The lysates containing intracellular *Salmonella* were collected, centrifuged and RNA was isolated from the bacterial pellets by a TRIzol-based method that yields both mRNA and sRNA. Briefly, the supernatant was discarded, the pellet was washed three times in 19% ethanol, 1% acidic phenol, re-suspended in the remaining liquid, transferred to a clean 1.5 mL Eppendorf tube and centrifuged at 20,000 × g at 4°C. The cell pellet was dissolved in 1 mL TRIzol (Invitrogen) on ice and transferred into a 2 mL heavy phase lock tube (5 Prime) into which 400 μL of chloroform was added and immediately mixed for 10 seconds. After incubation at room temperature for 2 minutes, the mixture was centrifuged at 20,000 × g for 15 minutes. The RNA present in the upper phase was transferred to a fresh tube, and precipitated by adding 450 μL of isopropanol and incubated at room temperature for 30 minutes. The precipitated RNA was then pelleted by centrifugation at 20,000 × g for 30 minutes. The pellet was washed in 350 μL ethanol (70%) and centrifuged at 20,000 × g for 10 minutes. The washed pellet was air-dried, re-suspended in RNase-free water by shaking (900 rpm) for 5 min in a heating block (65°C) (Peqlab Thriller) and stored at −80°C until cDNA library construction.

The integrity of RNA was verified using an Agilent Bioanalyzer 2100 and RNA concentrations were measured using the nanodrop spectrophotometer (Thermo Scientific) and the Qubit fluorometer (Invitrogen). Control RNA was isolated from bacterial cells grown in L- broth *in vitro* until ESP (see above). The infection process, RNA preparation, sequencing and analysis were conducted in duplicate to provide independent data from biological replicates.

### Library preparation and deep sequencing

The cDNA library preparation and Illumina sequencing was done by Vertis Biotechnologie AG (Freising, Germany). The total RNA obtained from the biological replicates of intra-macrophage was digested for 45 minutes with DNase I (Thermo Scientific) according to the manufacturer’s instructions. Ribosomal RNA was not depleted. RNA samples were fragmented with ultrasound (4 pulses of 30 sec at 4°C). The 3’ ends of RNA were then subjected to poly (A)-tailing using poly (A) polymerase. The RNA was then treated with TAP (Tobacco acid pyrophosphatase) to remove the pyrophosphate group from the 5’ end, prior to ligation with an RNA adapter. First strand cDNA synthesis was done with an oligo (dT) adapter and M-MLV-RNaseH-reverse transcriptase (Invitrogen), following PCR amplification of cDNA using high-fidelity DNA polymerase to a final concentration of approximately 20–30 ng μL^-1^. The cDNAs were purified using the Agencourt AMPure XP kit (Beckman Coulter Genomics), and analysed by capillary electrophoresis. The cDNA libraries were sequenced on an Illumina HiSeq 2000 system. For dRNA-seq, prior to cDNA preparation, an aliquot of the RNA samples were enriched for primary transcripts by treating with Terminator 5’-monophosphate dependent exonuclease (Epicentre; TEX) [22].

### Mapping of RNA-seq libraries and differential gene expression analysis

The sequence reads obtained from the different cDNA libraries were mapped against the 4/74 reference genome using the Segemehl software, with accuracy set to 100% [35,100]. The mapping coverage was increased by an iterative process that involved the sequential removal of any mismatched nucleotides from the 3’ end, and mapping the read against the 4/74 genome. This process was repeated until the individual sequence reads were accurately mapped to a single location on the chromosome, or until the length dropped below a minimum value of 20 nucleotides [17]. These uniquely-mapped reads were visualised with the Integrated Genome Browser (IGB) [101] and Jbrowse [102]. In total, 6 cDNA libraries (including the biological replicates of RNA-seq, dRNA-seq and RNA-seq of InSPI2 grown Δ*ssrA* & wild type *S*. Typhimurium 4/74) were generated.

The expression values of each gene were calculated from the uniquely-mapped reads using the Transcript per Million (TPM) approach [20,103]. TPM considers the transcripts to represent a mixture of two distributions of expressed and non-expressed genes, and so is ideal for the analysis of bacterial transcriptomic data. As this approach involves normalization to gene size and the total amount of genome-wide transcription, TPM values can be compared between genes and between growth conditions [20,103,104].

The threshold for expression of a gene was TPM value 10 [17]. Genes with TPM value ≤10 were considered to be “not expressed”. The differential expression of each gene or sRNA within macrophages was calculated against the ESP comparator as a fold change (macrophage versus ESP).

### Identification of *Salmonella* genes specifically up-regulated within macrophage

The average and standard deviation of RNA-seq data (TPM values) was calculated for each gene from the 20 in vitro growth conditions reported earlier [17]. For each gene, the standard deviation was multiplied by five-fold to define a broad expression range that captured all but the most extreme expression levels across the 20 conditions. To identify genes that were specifically up-regulated in macrophage, we selected a strict cut-off of 3-fold more highly expressed than five standard deviations above the mean expression value from the 20 conditions. In other words, macrophage specific gene = TPM > 3 x (average TPM in 20 conditions + 5σ). The genes that passed this cut-off are ‘not significantly expressed’ in any of the 20 *in vitro* conditions, are up-regulated within macrophages, and are listed in Fig 7A.

### The identification of transcriptional start sites (TSS)

A strict criterion was used to identify TSS, after visualization with the IGB browser [17]. Novel TSS were defined when a peak was enriched in the dRNA-seq data compared with the RNA-seq data in two biological replicates, and was located at the beginning of an expressed transcript.

The Promoter Usage Value (PUV) for each TSS was quantified by calculating the TPM for the first 10 nucleotides from the TSS towards the direction of transcription (from +1 to +10). The PUV values were classified as follows: (a) ‘Macrophage independent’ TSS have similar PUV in macrophages and at ESP (less than 2-fold up- or down-regulated); (b) ‘Macrophage up-regulated’ TSS are expressed at least 2-fold higher in macrophages relative to ESP; and (c) ‘Macrophage down-regulated’ TSS are expressed at least 2-fold less in macrophages relative to ESP.

### Confirmation of TSS by 5’ RACE

The 5’ RACE (rapid amplification of cDNA ends) was carried out with or without treatment by TAP using DNase I-digested total RNA isolated from the InSPI2 condition [105]. Gene specific amplification was done with the linker-specific primer JVO-0367 and gene specific reverse primers (Dataset 1 in S1 Table). TAP-enriched fragments were excised from an agarose gel, subcloned into a pTOPO vector (Invitrogen) and at least three clones were sequenced to validate individual TSS.

### Analysis of conservation of sRNAs between bacterial genomes

The sRNA nucleotide sequences from 4/74 were aligned against a set of bacterial genomes belonging to *Enterobacteriaceae* using GLSEARCH [106], and identical hits were extracted.

### Accession numbers

The RNA-seq data generated from this study are deposited at the NCBI GEO under the accession numbers GSM1462575 to GSM1462579, GSM1914919 and can be accessed at http://www.ncbi.nlm.nih.gov/geo/query/acc.cgi?acc=GSE59945.

## Supporting Information

S1 Fig 5’RACE data for four SPI2-located TSS. Agarose gels showing RT-PCR products generated from RNA treated with tobacco acid pyrophosphate (TAP; T+), a mock reaction (without TAP; T−) and of a control PCR reaction with 4/74 genomic DNA as template (ctrl.). The RNA was isolated from the InSPI2 growth condition (OD_600_ 0.3) [17]. Arrowheads mark the enriched band in TAP-treated samples (*ssaR* (A), *ssaM* (B), *ssaB* (C) and *ssaG* (D), indicating the cDNA of the respective primary RNA species. A DNA size marker is shown on the left [M, sizes in base pairs (bp)].
(PDF)

S2 Fig The intra-macrophage expression of functional categories of *Salmonella* genes. The Red and Blue bars indicate the percentage of genes of each functional category up-regulated or down-regulated inside macrophages versus ESP (Dataset 4 in S1 Table). The list of genes included in each functional category was obtained from the Kyoto Encyclopedia of Genes and Genomes, KEGG (http://www.genome.jp/kegg/).
(PDF)

S1 Table Dataset 1: Strains, growth conditions and PCR primers used in this study. Dataset 2: RNA-seq statistics. Dataset 3: List of all 3583 *Salmonella* TSS expressed during intra-macrophage survival. The expression level of TSS is shown as the Promoter Usage Value (PUV), calculated by determining the TPM of the first 10 nucleotides from the TSS, towards the direction of transcription (Materials and Methods). Dataset 4: The intra-macrophage expression levels of *Salmonella* Typhimurium 4/74 genes, shown as TPM values. The lowest TPM value is adjusted to 10, to allow statistical comparison. Dataset 5: Pathogenicity Island Expression Levels: The expression Levels for each PAI was calculated by averaging the fold change TPM (Macrophage versus ESP) of all the genes located within individual islands. Dataset 6: The intra-macrophage expression of Salmonella Typhimurium 4/74 regulatory genes. Data obtained from Dataset 4. Dataset 7: List of genes strongly induced within macrophages; represented as a comparison of expression in macrophages against the 20 *in vitro* conditions in Krӧger *et al*., 2013 [17]. This Dataset was used to generate Fig 7. Dataset 8: Candidate promoters for a *Salmonella*-based vaccine delivery system. Dataset 9: Intra-macrophage expression levels of 280 sRNAs of *Salmonella* Typhimurium 4/74. The lowest TPM value is adjusted to 10. This dataset was used to generate Fig 8. Dataset 10: Analysis of the conservation of 280 *Salmonella* sRNAs. The level of sequence identity of individual sRNAs was determined in 29 enterobacterial genomes with GLSEARCH. 1.00 indicates 100% identity. *Salmonella* specific sRNAs (Results and Discussion) are highlighted in green.
(XLSX)
