# Supplementary figures and images for: RNA-seq Brings New Insights to the Intra-Macrophage Transcriptome of Salmonella Typhimurium
Source: PLoS Pathog. 2015 Nov 12;11(11):e1005262. doi: 10.1371/journal.ppat.1005262 (PMC4643027; doi:10.1371/journal.ppat.1005262)

A.

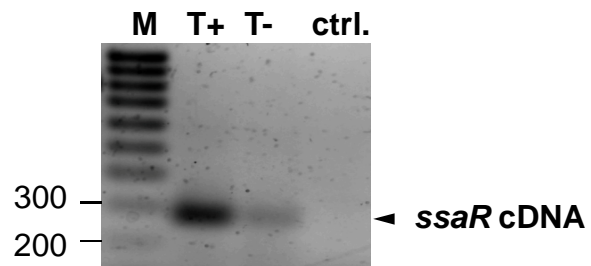

B.

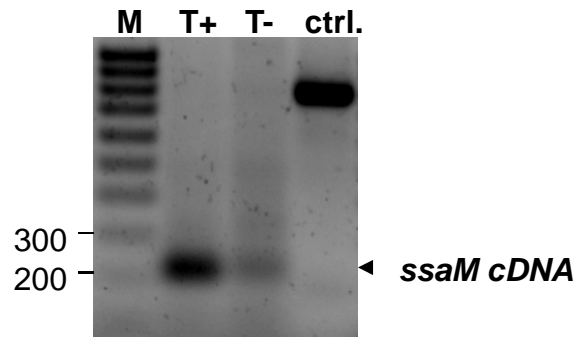

C.

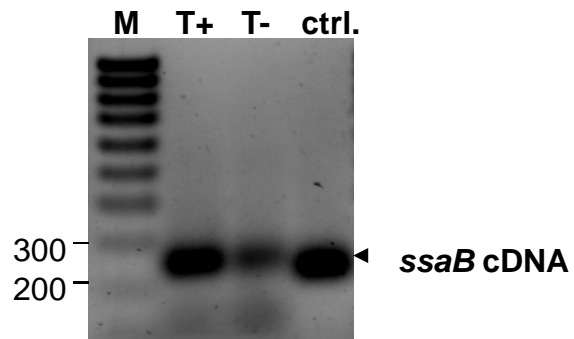

D.

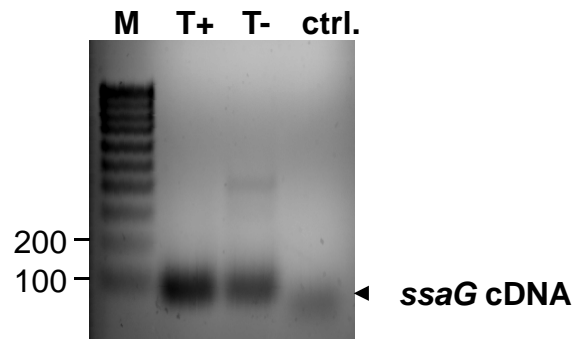

Supplement: S1 Fig — Agarose gels showing RT-PCR products generated from RNA treated with tobacco acid pyrophosphate (TAP; T+), a mock reaction (without TAP; T−) and of a control PCR reaction with 4/74 genomic DNA as template (ctrl.). The RNA was isolated from the InSPI2 growth condition (OD600 0.3) [17]. Arrowheads mark the enriched band in TAP-treated samples (ssaR (A), ssaM (B), ssaB (C) and ssaG (D), indicating the cDNA of the respective primary RNA species. A DNA size marker is shown on the left [M, sizes in base pairs (bp)]. (PDF) [file ppat.1005262.s001.pdf]

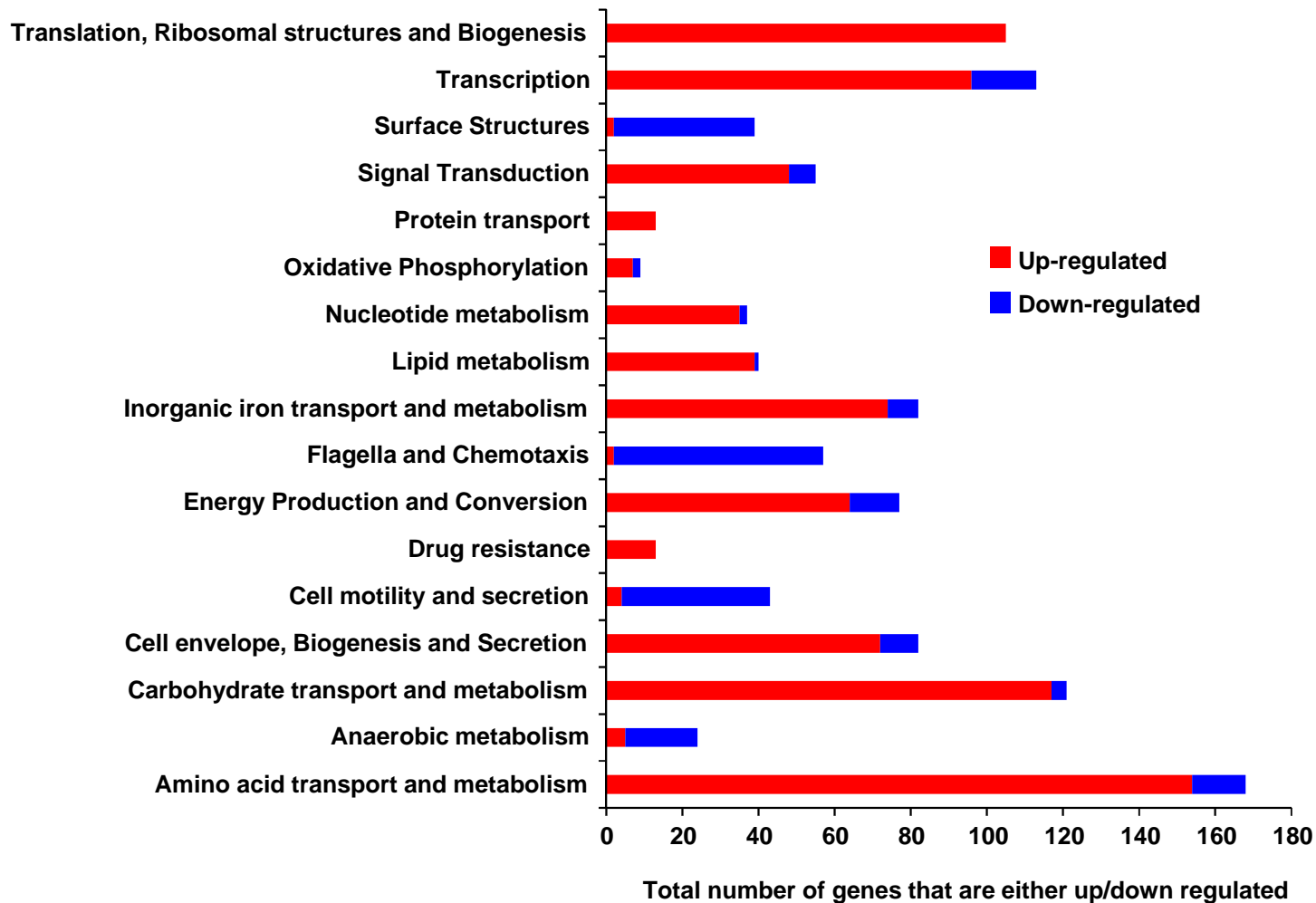

Supplement: S2 Fig — The Red and Blue bars indicate the percentage of genes of each functional category up-regulated or down-regulated inside macrophages versus ESP (Dataset 4 in S1 Table). The list of genes included in each functional category was obtained from the Kyoto Encyclopedia of Genes and Genomes, KEGG (http://www.genome.jp/kegg/). (PDF) [file ppat.1005262.s002.pdf]
